# Cesium polytungstates with blue-tint-tunable near-infrared absorption

**DOI:** 10.1039/d0ra00505c

**Published:** 2020-03-11

**Authors:** Satoshi Yoshio, Masao Wakabayashi, Kenji Adachi

**Affiliations:** Department of Computer-Aided Engineering, Sumitomo Metal Mining Co., Ltd. Ehime 792-0001 Japan satoshi.yoshio.w5@smm-g.com; Ichikawa Research Center, Sumitomo Metal Mining Co., Ltd. Ichikawa Chiba 272-8588 Japan

## Abstract

Revisiting Wöhler's method (1824), Cs-doped tungsten bronzes were synthesized by reducing Cs-polytungstate at high temperature, and were pulverized into nanoparticles for determining their optical properties. The high-temperature reduced Cs_4_W_11_O_35_ crystals absorbed strongly in the near-infrared, providing an improved luminous transparency with a less-bluish tint than normal Cs_0.32_WO_3−*y*_ synthesized in a reductive atmosphere. The high-temperature reduction caused an orthorhombic-to-hexagonal phase transformation and a nonmetal–metal transition, which was monitored by spectrophotometry, X-ray diffraction, and X-ray photoelectron spectroscopy measurements, assisted by a first-principles analysis using a DFT+U method. The high-temperature reduction of Cs_4_W_11_O_35_ is concluded to decrease the number of W deficiencies and produce oxygen vacancies, releasing both free and trapped electrons into the conduction band and thereby activating the near-infrared absorption. The comparatively narrow bandgap of Cs_4_W_11_O_35_ was identified as the origin of the less-bluish tint of the produced Cs tungsten bronzes.

## Abstract 2

Revisiting Wöhler's method (1824), Cs-doped tungsten bronzes were synthesized by reducing Cs-polytungstate at high temperature, and were pulverized into nanoparticles for determining their optical properties.

## Graphical abstract

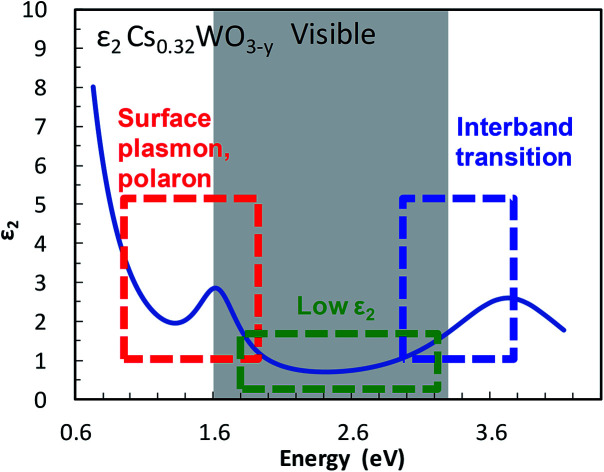

## I Introduction

Recently, hexagonal tungsten bronze (HTB) nanoparticles have demonstrated a high-level compatibility of luminous transparency and near-infrared (NIR) absorption,^1–3^ prompting their application in automotive and architectural windows, laser welding of resins, cancer therapies,^4,5^ and related technologies.

The origin of the strong NIR absorption in reduced tungsten oxides and tungsten bronze nanoparticles has been attributed to localized surface plasmon resonance (LSPR) of free electrons and polaronic excitation of trapped electrons.^6–16^ In Cs-doped HTB (Cs-HTB), recent analyses indicate that W-5d orbitals in the lower conduction band are occupied by the free and trapped electrons originating from Cs^+^ and oxygen vacancies (V_O_), respectively.^17,18,40^ The V_O_s have been analyzed to play a major role in the LSPR and polaronic excitation.^19,20^ On the other hand, the visible transparency of Cs-HTB nanoparticles, which is highest among the HTBs, has not been considered in detail, and its origin remains unclear. In addition, the slight bluish tint accompanying the transmitted light must be resolved in actual applications.

**Fig. 1:**
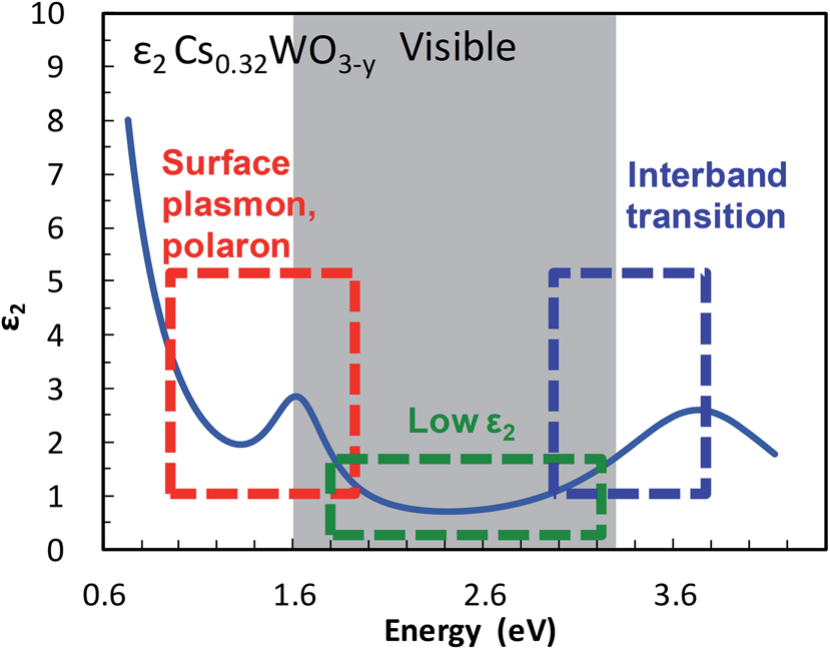
Transparency and color-determining factors shown in the imaginary component *ε*_2_ of the dielectric function of Cs_0.32_WO_3−*y*_.

The imaginary part *ε*_2_ of the dielectric function, which represents the absorption of a photon by an electron, is small at visible frequencies in Cs-HTB (see Fig. 1). The small *ε*_2_ is caused mainly by a wide band gap. Although the band gap of Cs-HTB is below 3.3 eV, the calculated electronic structure of Cs-HTB^17,18^ suggests that Fermi's selection rule prohibits certain electronic transitions such as W-5d → W-5d and O-2p → O-2p, which are involved in the hybridized W-5d/O-2p orbitals. This prohibition of certain transitions between metal-d and ligand-p orbitals further amplifies the visible transparency, enabling it even when the conduction and valence bands overlap in specific directions, as occurs in LaB_6_.^21^ The optical absorption of Cs-HTB nanoparticles is known large at both frequency ends of the visible region. On the blue side of the spectrum, the *ε*_2_ is enlarged by band-edge interband transition; on the red side, strong absorption occurs by LSPR and polaronic electronic transition.^13,20^ Therefore, the blue-tint problem in Cs-HTB nanoparticles reduces to the problem of controlling the band gap and the conduction-band electrons.

Cs-HTB is usually prepared by the solid-phase reaction in a reductive atmosphere. This study adopts a different approach that controls the band gap and V_O_-derived electrons. Magnéli and Blomberg^22^ summarized various methods of obtaining tungsten bronzes. The reduction of polytungstates (Cs_2_O·*n*WO_3_) in a steam of hydrogen gas flow was originally reported by Wöhler^23^ in 1824, but has been scarcely followed thereafter, so the synthesis product has not been reported in detail. Starting from a charge-neutral polytungstate (the Cs_4_W_11_O_35_ phase), we here synthesize a series of Cs-polytungstates (referred to as CPT) with a significantly reduced blue tint by the Wöhler's method. This reduction process involves the orthogonal-to-hexagonal phase transformation as well as the nonmetal–metal transition. The change in optical absorption is then related to these phase transitions.

## II Experimental

### II-1 Materials

Raw materials Cs_2_CO_3_ and WO_3_ of 99.9% purity were purchased from Fujifilm Wako Pure Chemical Corporation (Tokyo, Japan), and methyl isobutyl ketone (MIBK) of 99.5% purity was purchased from Kanto Chemical Co., Inc. (Tokyo, Japan).

As the starting material, Cs_4_W_11_O_35_ was selected because its Cs/W ratio was close to that of Cs_0.32_WO_3−*y*_. Cs_4_W_11_O_35_ was prepared by heating the Cs_2_CO_3_ and WO_3_ raw materials in air at 800 °C for 30 hours or at 850 °C for 5 hours, respectively. The X-ray diffraction (XRD) powder pattern revealed the primary Cs_4_W_11_O_35_ phase with a minutely mixed Cs_6_W_11_O_36_ phase.

### II-2 Powder and dispersion preparation

In the conventional gas flow reduction (GFR) method^19^ or the vacuum encapsulation method,^24^ the crystallization of Cs_0.32_WO_3−*y*_ and oxygen reduction proceed in parallel with the high-temperature reduction. On the other hand, when polytungstate crystals are reduced at high temperature, high crystallinity is expected from the early stages of reduction, which was considered as an advantage of the Wöhler's method. Thus, Cs_4_W_11_O_35_ was heated to 700–900 °C in an Ar gas flow in a tubular furnace. While maintaining the maximum temperature, the Ar gas injection was switched to a 1% H_2_–Ar gas, and the reduction was allowed to proceed for various durations. The reduced powder was cooled to 100 °C in an Ar gas flow, and removed from the furnace at room temperature.

The powder samples were blended with MIBK and a trace high-molecular-weight polymer dispersant at a powder-to-MIBK weight ratio of 1 : 50, and they were stirred in a paint shaker with 0.3 mmϕ zirconia beads to obtain a homogeneous dispersion. The particles were polished to a crystallite size of 23–35 nm by properly adjusting the polishing time that was monitored using the XRD Scherrer method. Details of the dispersion preparation method have been provided in ref. 25.

### II-3 Characterization and calculation methods

A series of powder samples was subjected to powder-XRD measurement using Cu-Kα radiation with an X'Pert-PRO/MPD apparatus (Spectris Co., Ltd., Tokyo, Japan). The diffraction angle was calibrated with a Si standard (NIST640e).

The nanoparticulate dispersions of the reduced products were further diluted with MIBK to a filler concentration of 0.05 wt%, injected into a transparent cell with a 1 mm optical path length, and its transmittance was measured with a U-4100 spectrophotometer (Hitachi High-Tech Corporation, Tokyo, Japan). The molar absorption coefficient was determined from the transmittance measurements. Visible light transmittance (VLT) was measured according to code JIS R3106. We measured solar transmittance according to JIS R3106 up to a wavelength of 2100 nm (ST21). It is defined by
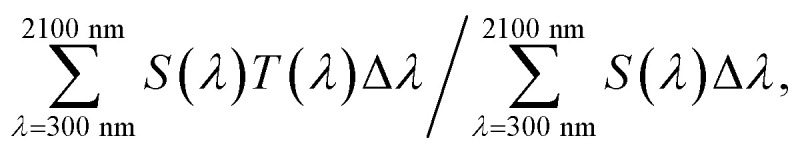
where *S*(*λ*) is the spectral distribution of the solar radiation, *T*(*λ*) is the spectral transmittance, and Δ*λ* is an increment of wavelength. Hunter's chromatic indices, L*, a*, and b* as well as RGB color indices were calculated according to JIS Z8701 and JIS Z8729.

Dispersions of neutral-toned In_2_O_3_ : Sn (ENAM Optoelectronic Material Co., Ltd., Funan, China; hereafter referred to as ITO) and bluish fully-reduced Cs-HTB prepared by the GFR process^19^ (hereafter referred to as CWO™) were prepared as color-tone references.

Powder size and morphology in those dispersions were observed using a transmission electron microscope (TEM) (HF-2200, Hitachi High-Tech Corp., Tokyo, Japan) operated at an acceleration voltage of 200 keV.

X-ray photoelectron spectroscopy (XPS) was performed using a Versa Probe II instrument (Ulvac-phi Inc., Chigasaki, Japan). As ion cleaning is known to reduce the sample surface,^19^ the measurements were performed on freshly as-produced powders. Okada *et al.*^19^ confirmed that Cs-HTB powders qualitatively give the same results as the bulk vacuum-cleaved surface. The precise position of the Fermi energy, *E*_F_, in the bottom of the conduction band was calibrated by evaporating Au on the second measurement.

First-principles calculations were performed in the Vienna *ab initio* simulation package (VASP),^26–29^ a calculation software based on plane-wave basis. Density functional theory with DFT+U method, which was confirmed to reproduce the optical properties of Cs_0.33_WO_3_ with high accuracy, was used.^18^ The +U values of the d orbital of W and the p orbital of O were 3.8 eV and 8.9 eV, respectively, as obtained in previous research.^18^

The optical properties of a material are defined by dielectric functions. Here, we applied the calculation method described in ref. 18. The dielectric function *ε* comprises a real part *ε*_1_ and an imaginary part *ε*_2_, as shown in eqn (1). The two parts are connected by the Kramers–Krönig relation shown in eqn (2). Briefly, if the imaginary part of the dielectric function can be accurately obtained up to the high-energy region, the whole dielectric function can be obtained by the Kramers–Krönig relation.

1 *ε*(*ω*) = *ε*_1_(*ω*) + i*ε*_2_(*ω*),

2
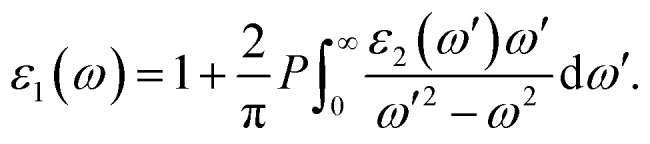
*ε*_2_ corresponds to the absorption of light energy by electrons, and comprises the Lorentz term (the contribution of interband transitions given by eqn (3)), and the Drude term (the contribution of free electrons). The former term, which is a 3 × 3 Cartesian tensor, is given by3
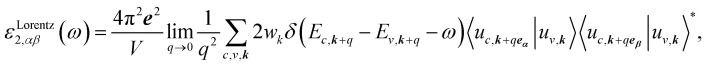
where *V* is the volume of the unit cell, and *c* and *v* are suffixes indicating the valence band and conduction band, respectively. *E* is the energy level, ***e*** is the unit vector with subscripts *α* and *β* denoting Cartesian coordinates, *u* is the periodic part of the orbit, and *w*_*k*_ is the weight of each set of *k* points.^30^

Cs-HTB is a conductor whose Fermi energy crosses the conduction band. Therefore, both the Lorentz and Drude terms are necessary. The Drude terms are functions of the plasma frequency *ω*_p_ (given by eqn (4)) and the relaxation time 
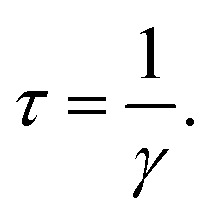
 They are expressed by eqn (5) and (6):4
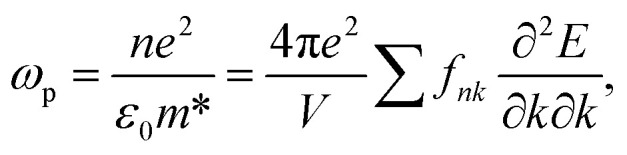
5
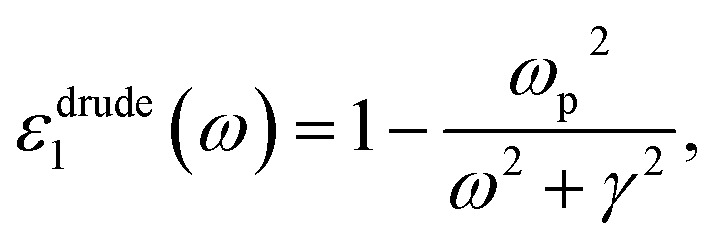
6
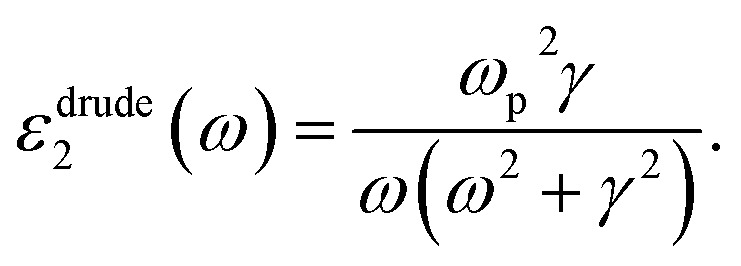


In eqn (4), *f*_*nk*_ is the Fermi–Dirac distribution function, *n* is the free electron density, *e* is the electron charge, *ε*_0_ is the dielectric constant in a vacuum, and m* is the effective mass of an electron.

Eqn (4) computes the plasma frequency from the band structure. The relaxation time indicates the effect of scattering caused by electron–phonon, electron–electron, and electron–lattice defect interactions. However, as *γ* does not appreciably affect the dielectric function, *ℏγ* was set to 0.1 eV in this study.

## III Results and discussion

### III-1 Optical properties of Cs-polytungstate

**Fig. 2:**
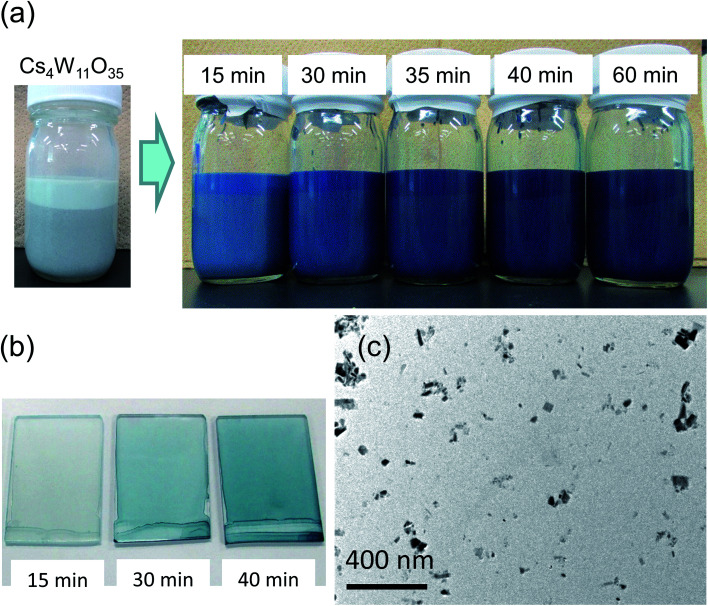
Color changes of (a) dispersed liquids in settled beads and (b) flow-coated glass plates of CPTs obtained by reducing Cs_4_W_11_O_35_ at 800 °C for various durations in a 1% H_2_–N_2_ gas flow. (c) TEM image of CPT dispersion reduced for 30 min.

Cs_4_W_11_O_35_ is a slightly greenish, almost white powder. Its MIBK dispersion is also white. Immediately upon reduction at 700–900 °C, a blue coloration occurred. As the reduction progressed, the color changed from sky blue to deeper blue to dark blue. Fig. 2(a) shows the color changes of the MIBK-dispersed CPT during Cs_4_W_11_O_35_ reduction at high temperature, and Fig. 2(b) shows their flow-coated films on a soda-lime glass. At reduction times of 15 minutes or less, the luminous color tone resembled that of ITO; after 40 minutes, it resembled that of CWO™. The bluish tone of the flow coating (using 0.05 wt% CPT dispersion) increased as the b* value decreased from 4.83 to −2.96 and then to −6.17. The TEM micrograph in Fig. 2(c) corresponds to the dispersed CPT nanoparticles reduced for 30 minutes at 800 °C and shows a typical image of CPT dispersions. The crystal shape is irregular because of the milling process, and the size scattered ranges from a few nm to 100 nm. The XRD Scherrer crystallite size was measured as 23–35 nm for every dispersion.

**Fig. 3:**
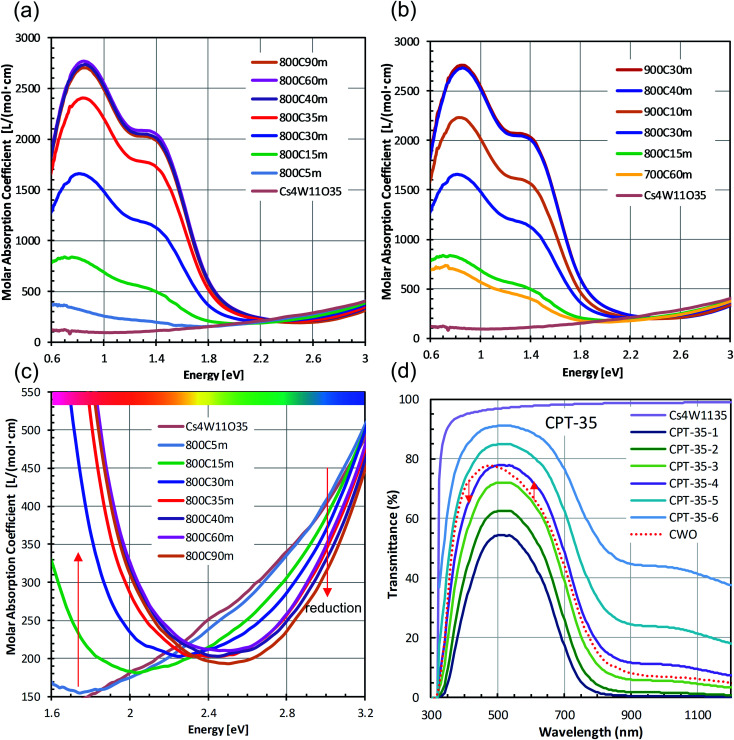
(a–c) Measured molar absorption coefficients of CPT obtained by reducing Cs_4_W_11_O_35_ at 700−900 °C for 15–60 min, and (d) transmittance profiles of the 800 °C – 35 min specimen, assuming Lambert–Beer's law.

Panels (a)–(c) of Fig. 3 plot the molar absorption coefficients of the CPT dispersions as functions of energy. Strong double-peaked absorptions appear in the 0.6–1.8 eV range. The low-energy (low-*E*) and high-energy (high-*E*) peaks are attributable to LSPR and polaronic absorptions, respectively, and the hidden intermediate LSPR peak arises from the crystal anisotropy.^20^ These absorptions strengthened with increasing reduction temperature and/or time. The absorption obtained by heating at 800 °C for 15 minutes was attained by heating at 700 °C for 1 hour. No absorption occurred by heating at 550 °C for 1 hour. Conversely, the state of 30 minutes at 800 °C was reached after only 10 minutes at 900 °C. Therefore, the following analysis concentrates on the treatment at 800 °C.

Fig. 3(c) is an enlarged view of the visible-range absorption curves in Fig. 3(a). The profile of Cs_4_W_11_O_35_ monotonously decreased from blue to red. At the blue side of the spectrum, the absorption decreased with increasing reduction time. Conversely, at the red side of the spectrum, the absorption remarkably increased with increasing LSPR absorption over time. Fig. 3(d) shows the series of transmission profiles of the samples with various CPT concentrations in dispersion, reduced for 35 minutes at 800 °C. The curves were derived from the molar absorption coefficient and the Lambert–Beer equation. Relative to the profile of CWO™ (fully-reduced Cs-HTB produced by the normal route of GFR synthesis^19^), the CPT transmission was reduced in the blue regions and increased in the red regions. Accordingly, the CPT series exhibits a more tint-neutralized profile than CWO™.

Here, it is noted that the optical profiles of the CPT dispersions shown above are highly consistent in terms of the degree of reduction even though certain irregularities in the size and morphology of the nanoparticles are observed in TEM images. This trend arises from the ensemble inhomogeneity effect of nanoparticle dispersions^20,25^ that generally broadens the absorption band and red-shifts the absorption peak, thereby masking the variation in individual Mie scattered waves due to differences in shape and physical properties.

**Fig. 4:**
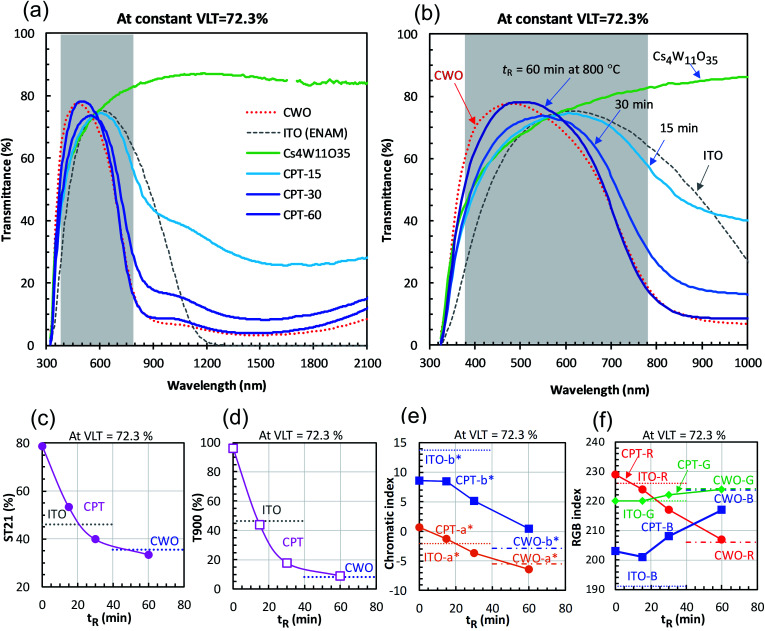
(a and b) Transmittance profiles of CPT and (for comparison) fully-reduced CWO and ITO at constant visible light transmittance (VLT = 72.3%). The profile of Cs_4_W_11_O_35_ at VLT = 72.3% is also shown. Reduction time dependence of (c) solar transmittance, (d) transmittance at 900 nm, (e) CIE color indices, and (f) RGB indices.

Fig. 4 shows the changes in transmittance profiles, solar transmittance, CIE color indices, and RGB color indices of the dispersions reduced at 800 °C. For comparison, the evaluation results of neutral-colored ITO dispersion (manufactured by ENAM) and blue-tinted CWO™ dispersion synthesized by the normal route are also shown. As these optical indices depend on the transmittance value, the visible light transmittance (VLT) in this comparison was fixed at 72.3%. The corresponding L* range was 88.12 ± 0.22.

The reduction of Cs_4_W_11_O_35_ was accompanied by a large NIR absorption around 1500 nm (Fig. 4(a)), almost equaling that of CWO™ after 60 minutes' reduction. Owing to polaronic excitation, the absorption around 850 nm was characteristically stronger in CPT than in ITO. In the visible region (Fig. 4(b)), the transmittance of CPT in the blue region (relative to that of ITO) gradually increased with reduction time, approaching that of CWO™ at 60 min. In the red region, the transmittance of CPT sharply decreased owing to neighboring NIR absorption. The transmittance in this region was comparable to that of ITO at 15 min, and approached that of CWO™ at 60 min.

The NIR shielding effect increased with reduction time. After 20 minutes of reduction, the solar transmittance (ST21) was lower in CPT than in ITO (Fig. 4(c)); after 50 minutes, the transmittances in CPT and CWO™ were equal. Similarly, the CPT transmittance in the near-infrared region (T900, *λ* = 900 nm) was below that of ITO at 15 minutes and reached the CWO™ level at 60 min (Fig. 4(d)). That is, the solar shielding property was higher in CPT than in ITO after 20 minutes of reduction. This high shielding is attributable to high absorption of CPT in the 700–1000 nm region, where the solar intensity is high.

Fig. 4(e) shows the changes in the CIE color indices (a* and b*) as functions of reducing time. As the reducing time increased, a* changed from magenta to green while b* changed steeply from yellow to blue. Even after 60 minutes of reduction, the b* of CPT remained positive, indicating a weaker bluish tone than in CWO™ (with b* = −2.89). Similarly, the R (red) index of CPT in the RGB color system linearly decreased with reduction time, whereas the B (blue) index increased with reduction time after a brief decline at 15 minutes. In summary, although the bluish tint in white Cs_4_W_11_O_35_ generally strengthened with reduction time, a less-bluish color tone was compatible with the high solar shielding effect of CWO™ in the 30−60 min reduction time at 800 °C.

### III-2 Structural changes during Cs_4_W_11_O_35_ reduction

**Fig. 5:**
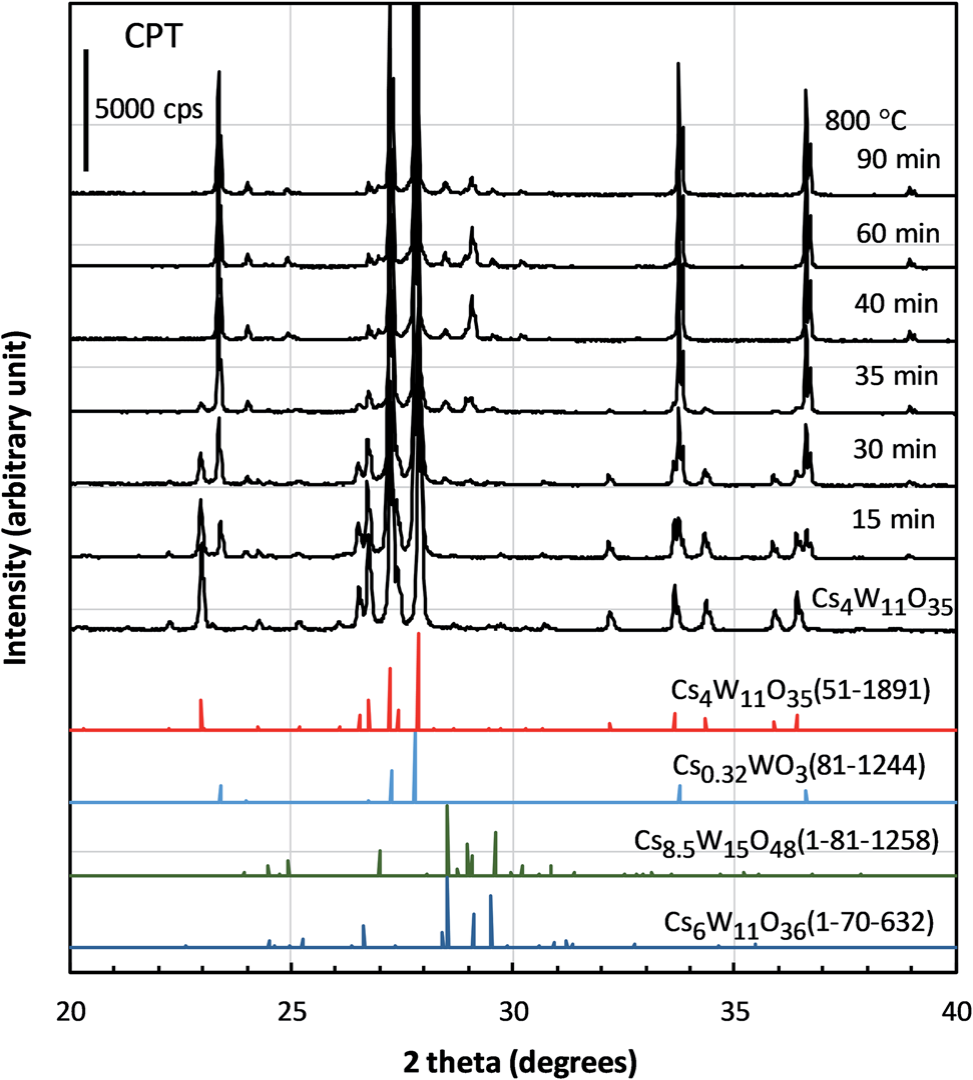
XRD profiles of Cs_4_W_11_O_35_ and the specimens reduced at 800 °C for different durations. Also shown are the profiles of Cs_4_W_11_O_35_ (51-1891), Cs_0.32_WO_3_ (81-1244), Cs_8.5_W_15_O_48_ (01-81-1258), and Cs_6_W_11_O_36_ (01-70-632) extracted from the International Centre for Diffraction Data.

The structural factors responsible for the optical characteristics in Fig. 2–4 are presented. Fig. 5 shows the changing XRD powder patterns of Cs_4_W_11_O_35_ reduced at 800 °C. The as-produced Cs_4_W_11_O_35_ exhibited almost a single-phase pattern, consistent with an orthorhombic phase of space group *Pbc*2_1_ as reported by Solodovnikov *et al.*^31^ As the reduction time increased, the reflections of the orthorhombic Cs_4_W_11_O_35_ phase gradually decreased (almost disappearing at 40 minutes), while the reflections of the hexagonal Cs_0.32_WO_3_ phase^32^ gradually increased and dominated after 30 minutes. The space group of the hexagonal phase is *P*6_3_/*mcm* as originally determined by Magnéli^33^ and as reported in recent works.^19,32,34^ The excess peaks around 2*θ* = 28–31° that appeared in the later stage of reduction matched only Cs_8.5_W_15_O_48_ (space group: *R*3̄*m*) or Cs_6_W_11_O_36_ (space group: *Aa*) among the ICDD data base. Although the intensity distributions do not completely match those of Cs-rich Cs_8.5_W_15_O_48_ or Cs_6_W_11_O_36_, speculation exists that some modifications of these phases precipitated because the Cs composition becomes excessive with the growth of W-rich Cs_0.32_WO_3_ = Cs_4_W_12_O_36_ phase. From the initial stage to 40 minutes of reduction, the powder-XRD characterization revealed a mixed-phase CPT comprising Cs_4_W_11_O_35_ and Cs_0.32_WO_3_.

**Fig. 6:**
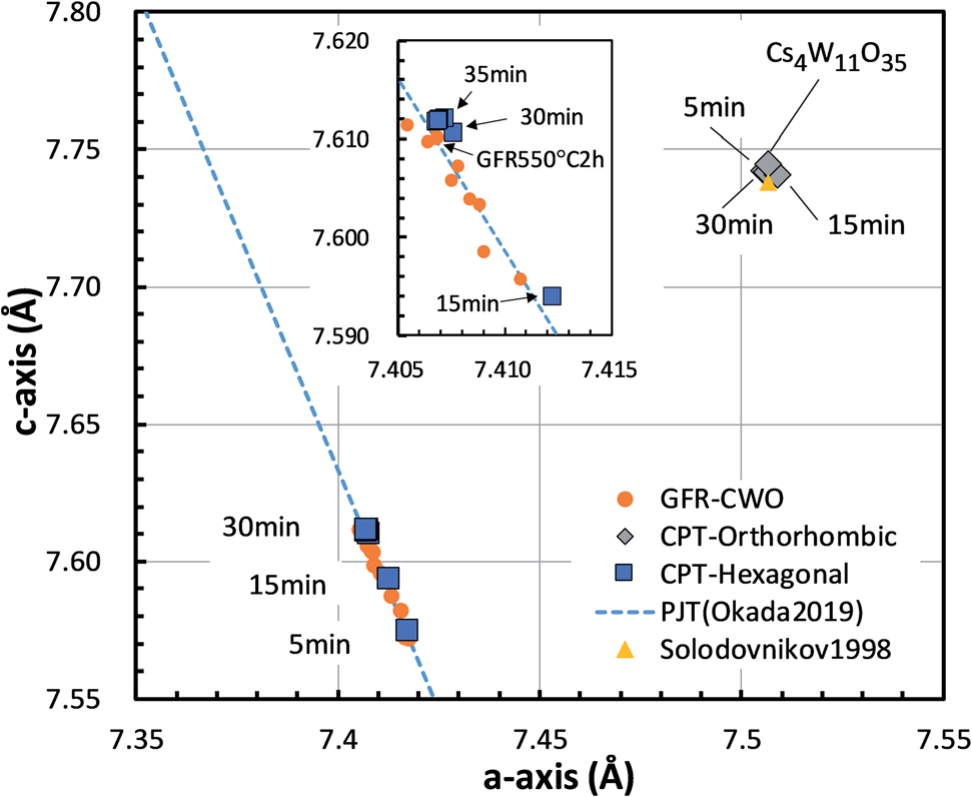
Lattice constants of the Cs_0.32_WO_3_ (CPT-hexagonal) and Cs_4_W_11_O_35_ (CPT-orthorhombic) portions in CPTs formed by reduction at 800 °C for various times. The *c*-axis *versus a*-axis is plotted in the hexagonal basis. The plot is compared with those of Cs_4_W_11_O_35_ by Solodovnikov *et al.*^31^ and Cs_0.32_WO_3−*y*_ produced by gas flow reduction (GFR-CWO) at 550 °C for varying reduction times.^19^ The dashed line shows the pseudo Jahn–Teller-induced variation by Okada *et al.*^19^

The lattice constants of the hexagonal components were obtained by the Rietveld method assuming *P*6_3_/*mcm*. The lattice constants of the orthorhombic phase were determined by the Pawley method assuming *Pbc*2_1_ because the precise atomic positions were undetermined. Fig. 6 shows the changing lattice constants with increasing reduction time in the hexagonal *a*–*c* space. The Cs_4_W_11_O_35_ lattice is classified as orthorhombic, but in the model of Solodovnikov *et al.*,^31^ it can also be regarded as a modified hexagonal lattice with W- and O-deficient planes periodically inserted in {020}_ORTH_//{100}_HEX_. Here the orthorhombic lattice constants were converted to hexagonal lattice constants by the formula:^31^7
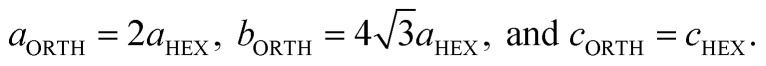


As the reduction proceeded, the CPT was decomposed into orthorhombic and hexagonal fractions. The hexagonal components were separated at positions of smaller *a* and *c* than the orthorhombic component. At later reduction times, the hexagonal *a*-axis and *c*-axis contracted and elongated, respectively, shifting to positions close to those of fully-reduced CWO™, where they saturated. In recent experiments,^17,19^ we showed that as the Cs and/or V_O_ concentration increases, the lattice constants of GFR-derived CWO™ move along the dashed line in Fig. 6 to the upper left. This behavior is triggered by electron donation to W-5d in the conduction band, which destabilizes the pseudo Jahn–Teller (PJT) distortion.^17,35^ The lattice constants of the GFR-derived CWO™ were constant after two hours' reduction at 550 °C around the point labeled ‘GFR550°C2h’ in Fig. 6. When the reduction time exceeded four hours at 550 °C, WO_2_ and W began precipitating. In the present reduction of Cs_4_W_11_O_35_, the lattice constants similarly converged toward the full reduction saturation point as the reduction proceeded. This behavior can reasonably be attributed to destabilization of the PJT distortion as proposed in Okada *et al.*^19^ Clearly, the reduction process injects electrons into the W-5d orbitals of the conduction band.

### III-3 Electronic changes during Cs_4_W_11_O_35_ reduction

**Fig. 7:**
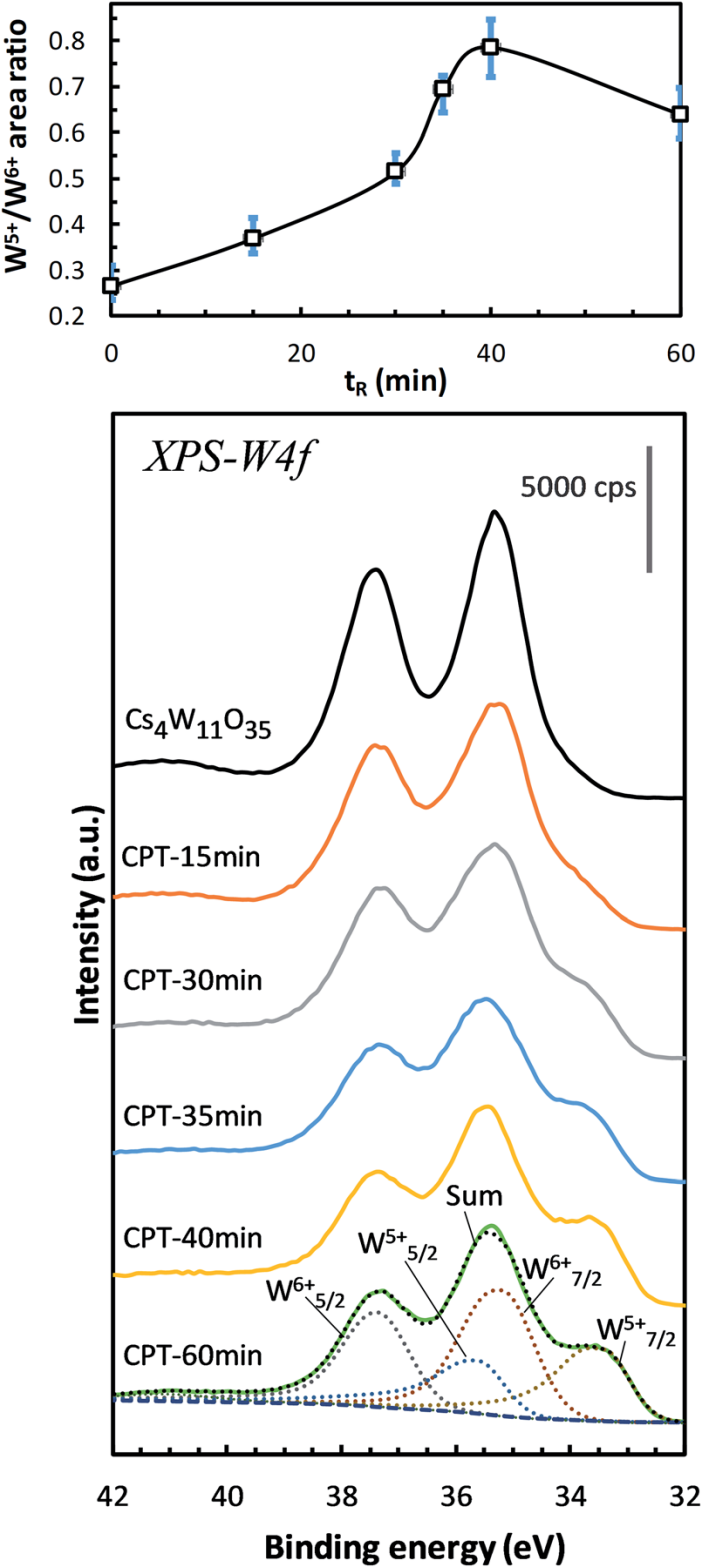
W-4f spectra of Cs_4_W_11_O_35_ and CPTs (15 ≤ *t*_R_ ≤ 60 min), showing the increasing intensities of W^5+^ relative to those of W^6+^*versus* reducing time *t*_R_. The growing W^5+^ indicates the production of oxygen vacancies.^19^

This subsection examines the factors in electronic structures that explain the optical properties shown in Fig. 2–4. Fig. 7 shows the changes in the W-4f XPS spectrum with increasing reduction time. As the reduction proceeded, the W_7/2_^5+^ peak at around 33.5 eV increased while the W_7/2_^6+^ peak at around 35.2 eV decreased. When plotted as a function of reduction time, the peak area ratio of W^5+^/W^6+^ increased to its maximum at 40 minutes, then decreased at 60 minutes. Similar changes have been observed in GFR-derived samples.^19^ Okada *et al.* determined the amount of V_O_s by chemical analysis, and concluded that V_O_s produces W^5+^ that supplies electrons to the conduction band, thus destabilizing the PJT distortion. Machida *et al.*^20^ analytically decomposed the optical absorption peaks into three components: a polaron peak and two anisotropic plasmon peaks. After extrapolating the intensity change of the polaronic component to zero reduction time, they found that the polaron component vanishes in the absence of V_O_. Furthermore, in first-principles calculations, Yoshio and Adachi^18^ showed that the 1.6 eV peak of *ε*_2_ is correlated with polaronic absorption and requires the presence of V_O_. They also reported that when V_O_ is generated, charges are produced at neighboring W^6+^ to generate a set of W^5+^s. Therefore, the W^5+^ increase in Fig. 7 is attributable to the generation and increase of V_O_s by the reduction of Cs_4_W_11_O_35_.

**Fig. 8:**
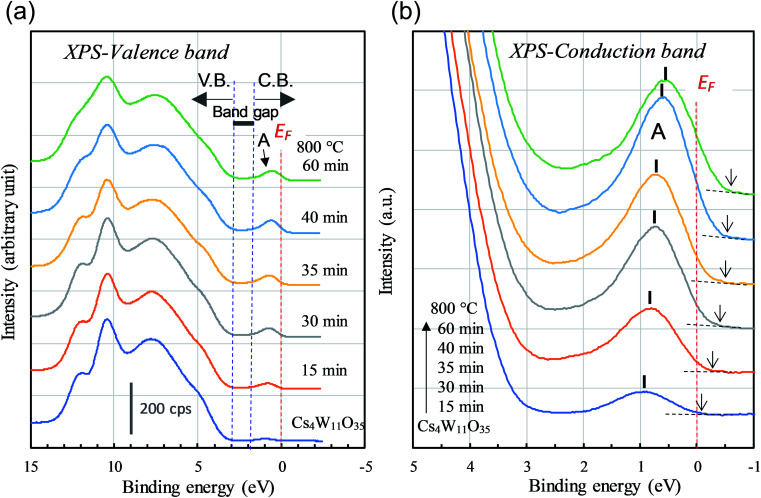
XPS (a) valence- and (b) conduction-band spectra of Cs_4_W_11_O_35_ and CPTs as the reducing time *t*_R_ varied from 15 to 60 min. The number of conduction-band electrons increases with *t*_R_.

Next, we observe the XPS valence band. As shown in Fig. 8(a), the reduction of Cs_4_W_11_O_35_ decreased the broad O-2p peaked at 8 eV and 10 eV below the band gap. The weak peak at the bottom of the conduction band (labeled “A”) grew as the reduction progressed. Peak A corresponds to the contribution of free and localized electrons^17,18^ existing in W-5d in the lower conduction band. In the non-conductor Cs_4_W_11_O_35_, peak A was caused by a slight reduction at the sample surface under the intense X-rays, which may be disregarded. At reduction times exceeding 40 min, the lower conduction band should be fully populated with free electrons, in analogous to fully-reduced CWO™; indeed, the *E*_F_ crosses peak A at its half maximum. However, where the reduction was small, the peak height gradually decreased while simultaneously shifting toward the high-*E*_B_ side (valence-band side) with nearly zero density of states at *E*_F_. This phenomenon is considered to arise from the Coulomb gap.^36^ In the archetype tungsten bronze, Na_*x*_WO_3_, the decreasing electron density at *E*_F_ with decreasing Na content has been observed as the Coulomb gap.^37–39^ As more electrons populate the conduction band, the Coulomb gap decreases. Because the Coulomb gap is caused by long-range interactions among trapped electrons, its presence in the CPT system further evidences electron-trapping in this system.

The above observations imply that when Cs_4_W_11_O_35_ is reduced at high temperatures, the electrons are donated to the conduction band by eliminating the W-defected planes caused by the orthorhombic-to-hexagonal transformation. Meanwhile, the parallel generation of V_O_ provides additional electrons to the conduction band. The electrons generated by both processes boost the NIR absorption capability.

Finally, the observed changes in electronic structures were examined by first-principles calculations.

**Fig. 9:**
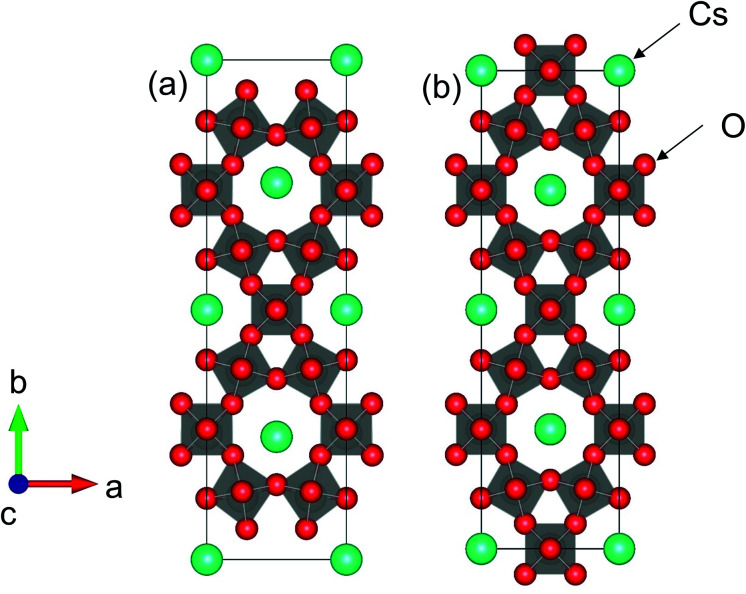
Orthorhombic (001) projections of (a) Cs_4_W_11_O_35_ and (b) Cs_4_W_12_O_36_ (Cs_0.33_WO_3_).

Fig. 9 shows the crystal structures of (a) Cs_4_W_11_O_35_ and (b) Cs_4_W_12_O_36_. The structure in Fig. 9(b) is equivalent to Cs_0.33_WO_3_ reoriented in an orthorhombic basis for structural comparison with Cs_4_W_11_O_35_. The structure in Fig. 9(a) is that of Fig. 9(b) with the removal of W and O on the (010) plane. The first-principles calculations considered the terminal structures of Fig. 9(a) and (b), and the W-deficient and O-deficient versions of the structure in Fig. 9(b).

**Fig. 10:**
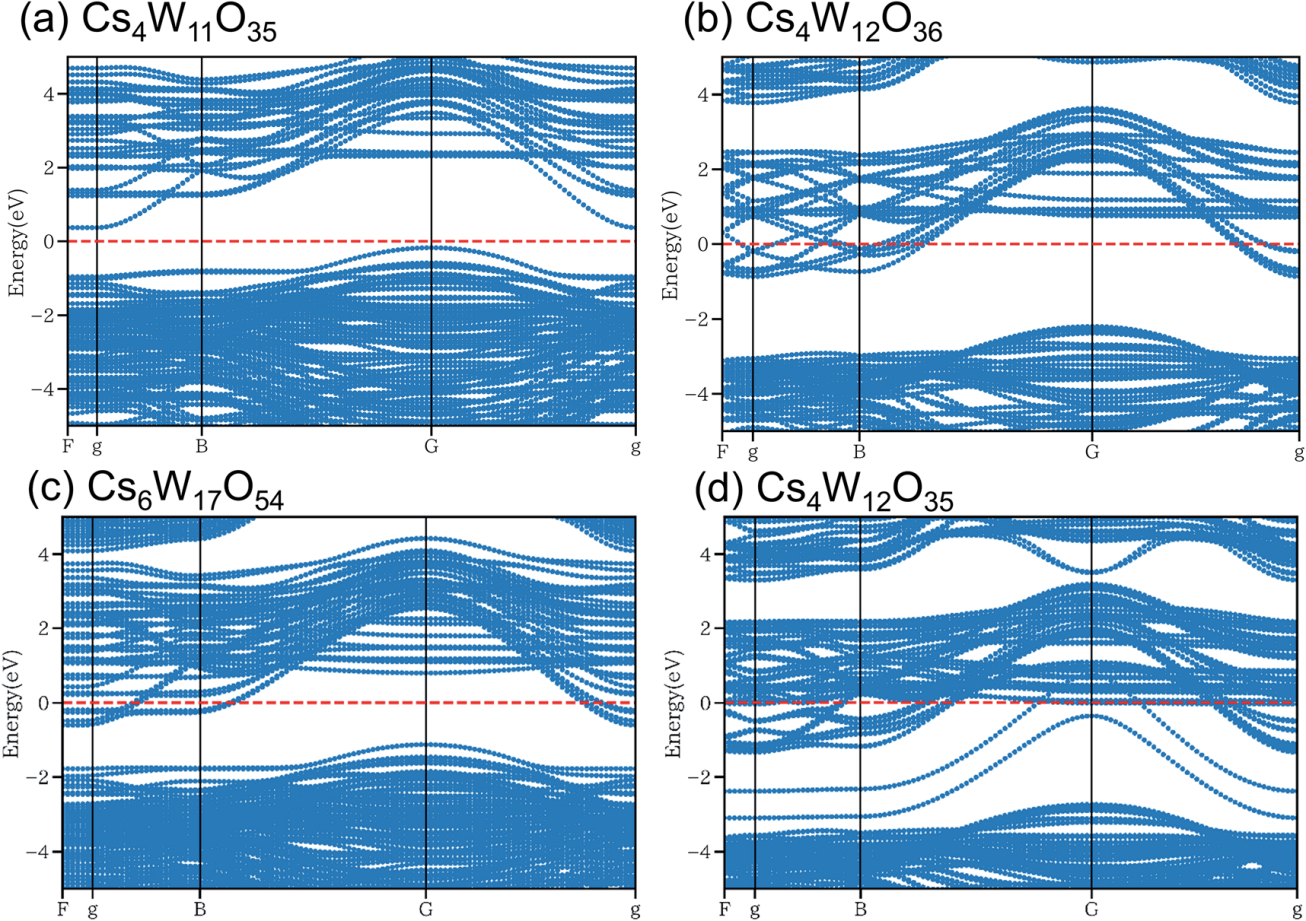
Band structures of (a) Cs_4_W_11_O_35_, (b) Cs_4_W_12_O_36_=Cs_0.33_WO_3_, (c) Cs_6_W_17_O_54_ and (d) Cs_4_W_12_O_35_ calculated by DFT+U method. Red dashed lines denote the Fermi energy.

As shown in Fig. 10, the conduction and valence bands of Cs_4_W_11_O_35_ and Cs_4_W_12_O_36_ have similar overall dispersion, although the position of *E*_F_ is different. The *E*_F_ locates in the band gap of the former, and in the lower conduction band of the latter. Therefore, Cs_4_W_11_O_35_ is a non-conductor and Cs_4_W_12_O_36_ is a conductor. With fully-arranged W and O atoms and a complete hexagonal WO_3_ network, Cs_4_W_12_O_36_ can inject its Cs^+^ electrons into the W-5d orbitals, conferring a conducting property.^6,17,18^

In the Cs_6_W_17_O_54_ of Fig. 10(c), one W has been removed from Cs_6_W_18_O_54_, the cell of which is 1.5 times larger along the *b*-axis direction of Cs_4_W_12_O_36_. Although the charge is formally neutral in the chemical formula Cs_6_W_17_O_54_ = 3Cs_2_O·17WO_3_, the material is considered as a conductor because *E*_F_ crosses the lower conduction band. When the W defect (V_W_) was created, many localized levels were generated and the band gap was altered, implying an intrinsic change in the band structure. In contrast, when Cs is varied in Cs_*x*_WO_3_ (not shown here), the band structure is almost unchanged (only the *E*_F_ position changes).^18^

In the structure of Fig. 10(d), one O has been removed from the Cs–O plane of Cs_4_W_12_O_36_. Additional electron orbitals generated in the band gap were partially localized in specific directions. Essentially, the same result was reported in a more detailed calculation^18^ that included V_O_s residing at multiple sites in both the Cs–O and W–O planes. When V_O_ is generated, the band structure changes as observed in V_W_.

According to the XRD observations, the reduction process induced a structural transformation from Cs_4_W_11_O_35_ to Cs_4_W_12_O_36_. The starting Cs_4_W_11_O_35_ contained fewer W and O atoms than Cs_4_W_12_O_36_. In part, these W and O deficiencies are probably inherited by the hexagonal CPT. As the reduction proceeded, the amounts of V_W_ and V_O_ decreased and increased, respectively. The V_W_ decrease corresponded to the changing band structures in Fig. 10(a) → (c) → (b), whereas the V_O_ increase corresponded to the changes in Fig. 10(a) → (b) → (d). In both cases, the *E*_F_ was displaced into the conduction band and the number of conduction electrons increased. Therefore, peak A in the XPS spectrum grew with increasing reduction time (Fig. 8), and the PJT destabilization shifted the lattice constants (Fig. 6).

**Fig. 11:**
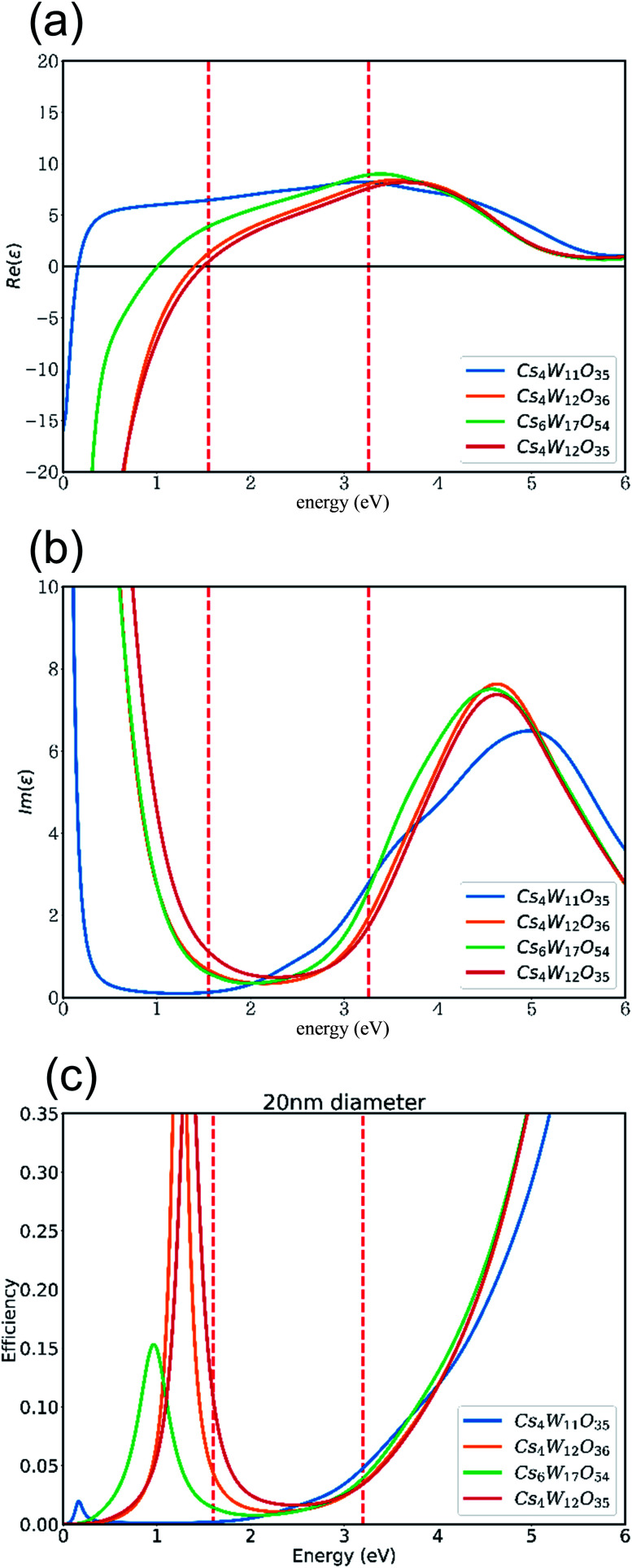
(a) Real and (b) imaginary parts of the dielectric functions, and (c) the Mie scattering efficiency, of 20 nm-diameter particles of Cs_4_W_11_O_35_, Cs_4_W_12_O_36_=Cs_0.33_WO_3_, Cs_6_W_17_O_54_, and Cs_4_W_12_O_35_.

Based on these band structures, the dielectric functions (including the Drude term) were calculated, and their real and imaginary parts are plotted in panels (a) and (b) of Fig. 11, respectively. At *ε*_1_ = 0 in Fig. 11(a), the screened plasma frequency (*Ω*_sp_) is seen to increase in the order of Cs_4_W_11_O_35_ → Cs_6_W_17_O_54_ → Cs_4_W_12_O_36_ → Cs_4_W_12_O_35_. The NIR absorption is expected to increase in the same order, although experimentally, the disappearance of V_W_ accompanies the generation of V_O_.

As clarified in Fig. 11(b), the imaginary part *ε*_2_ of CPT and CWO™ is generally small in the visible-light region, as expected. This result explains the high visible transparency of these materials. At 3.3 eV in the blue region of the spectrum, the interband transition dictates the optical absorption. Thus, the Cs_4_W_11_O_35_ absorption is enlarged by the narrow band gap. On the other hand, at 1.6 eV in the red region, the Cs_4_W_12_O_35_ absorption is enlarged by the high *Ω*_sp_. Therefore, the transmitted light in the red region should also decrease in the order of increasing *Ω*_sp_ as stated above.

Fig. 11(c) shows the Mie scattering efficiency of a 20 nm-sized spherical particle calculated using these dielectric functions. Comparing panels (b) and (c) of Fig. 11 with the experimentally observed curves of molar absorption coefficients (Fig. 3(c)), one finds that the dielectric functions well reproduce the optical change in the visible region induced by the reduction process. Notably, the V_W_ increased the light absorption at the high-*E* side. The V_W_ amount is expected to be lower in the GFR-method than in Wöhler's method, but the V_O_ amount should be comparable in both methods. The results in Fig. 11 show that V_W_ increased and decreased the absorption at the high- and low-*E* sides, respectively, causing a more neutral shift in color tone than in normal CWO™.

According to these calculations, the less-bluish CPT obtained in this study than in normal-route CWO™ was attributed to the narrow band gap of Cs_4_W_11_O_35_. CPT containing V_W_ and V_O_ can be categorized as tungsten bronze based on its structural and electronic features, but its energy-band structure slightly differs from that of CWO™. From an application perspective, the band gap of CPT and the amount of electrons injected into the conduction band can be controlled by adjusting the high-temperature reduction condition of polytungstates, thereby achieving the desired NIR absorption effect with a low bluish color tint.

## IV Conclusion

Revisiting Wöhler's method, we obtained Cs-doped tungsten bronze by reducing a Cs-polytungstate at high temperature. When orthorhombic Cs_4_W_11_O_35_ was reduced at 700–900 °C, the NIR absorption and visual bluishness increased with reduction time. The structural phase transformation to hexagonal Cs_0.32_WO_3−*y*_ was observed to occur that accompanied the decrease in W defects. The linear change in the *c*-axis *versus a*-axis lattice constants of the hexagonal component was explained by destabilization of the pseudo Jahn–Teller distortion. Eventually, the lattice constants converged to those of fully-reduced CWO™ produced *via* the normal route. The XPS W^5+^ 4f signal increased as the reduction proceeded, confirming the formation of V_O_. In the early reduction stages, the Coulomb gap was observed near the *E*_F_ of the Cs-polytungstates, indicating the presence of trapped electrons. First-principles calculations confirmed that the decrease in W deficiency and/or the increase in O deficiency increased the *E*_F_ from the band gap to the conduction band. As Cs_4_W_11_O_35_ has a comparatively narrow band gap and absorbs blue light, the reduced Cs-polytungstate decreased the transmission of blue wavelengths, achieving a less-bluish tone than fully-reduced CWO™ obtained by the normal route. A nonmetal–metal transition occurred when the Cs^+^-derived and V_O_-derived electrons were donated to the W-5d orbitals in the conduction band, during the formation of the hexagonal lattice and the generation of V_O_s by the high-temperature reduction annealing.

## Conflicts of interest

There are no conflicts of interest to declare.

## Supplementary Material
